# Investigating war trauma, its effects, and associated risk factors on anxiety among high school students in Woldia town, northeast Ethiopia, 2022

**DOI:** 10.3389/fpsyt.2024.1368285

**Published:** 2024-07-10

**Authors:** Mulat Awoke Kassa, Tamrat Anbesaw, Girum Nakie, Mamaru Melkam, Molla Azmeraw, Elsabet Gezmu Semagn, Biruk Beletew Abate

**Affiliations:** ^1^ Department of Nursing, College of Health Sciences, Woldia University, Woldia, Ethiopia; ^2^ Department of Psychiatry, College of Medicine and Health Sciences, Wollo University, Dessie, Ethiopia; ^3^ Department of Psychiatry, College of Medicine and Health Sciences, University of Gondar, Gondar, Ethiopia; ^4^ Department of Veterinary Parasitology, College of Veterinary Medicine, Wollo University, Dessie, Ethiopia

**Keywords:** anxiety, high school students, adolescents, war, conflict, Ethiopia

## Abstract

**Background:**

Anxiety symptoms are the most frequent mental health problems in the world, and it is a serious public health concern, especially among adolescents, because if left untreated, adolescent anxiety can have a number of detrimental effects, including lower academic performance, strained relationships with friends and family, substance addiction, thoughts of suicide and homicide, and trouble finding work. While this vulnerability is concerning in all situations, it is particularly critical in armed conflict areas. Ethiopia is one of the most recent war-affected countries, but to the best of our knowledge, limited studies focused on adolescents in this context. Therefore, this study assessed the experience of war trauma and its effects on anxiety symptoms among high school students in Woldia town, Ethiopia.

**Objective:**

We assessed the experience of war trauma and its effects on anxiety among high school students, as well as associated risk factors, in war-affected areas in Woldia town, northeast Ethiopia.

**Methods:**

A cross-sectional study design was conducted from May 23 to June 08 2022. Data were collected from high school students in Woldia town. Bivariable and multivariable logistic regression analyses were used to identify independent factors associated with anxiety.

**Results:**

A total of 624 out of 660 students participated in the study (94.5% response rate). The prevalence of anxiety among high school students in Woldia town was 39.7%. In the multivariable analysis, having depression (AOR = 9.24, 95% CI: 6.27, 13.64), witnessing the murder of family/friends (AOR = 1.93, 95% CI: 1.05, 3.57), being of female sex (AOR=1.59, 95% CI: 1.08, 2.36), and having a family history of mental illness (AOR=1.69, 95% CI: 1.00, 2.85) were factors significantly associated with anxiety.

**Conclusions and recommendations:**

The prevalence of anxiety in this study was approximately two in five high school students. Therefore, we recommend that the Ministry of Education collaborate with the Ministry of Health to expand and implement mental health services in high schools to promote the wellbeing of students for the prevention of anxiety.

## Introduction

Adolescence is a critical stage of life marked by significant and quick changes in one’s physical, intellectual, emotional, and psychological makeup (1), as well as the emergence of potentially dangerous or beneficial lifelong habits. Half of all mental health illnesses that completely develop in adulthood, according to the WHO, start before the age of 14 (2).

Anxiety is a normal human emotion characterized by various responses (e.g., behavioral, affective, and cognitive) to perceived threats (3). However, when such reactions result in a great deal of distress or are out of proportion to the perceived source of stress, anxiety may be deemed excessive or pathological (4). Anxiety is defined by its physical symptoms such as palpations, trembling, and shortness of breath, in addition to subjective feelings, thoughts, and observable fight-flight-freeze behavior (5). The Diagnostic and Statistical Manual of Mental Disorders (DSM-5) lists restlessness or feeling tense or agitated, easily becoming fatigued, trouble focusing or losing one’s train of thought, irritability, muscle tension, and sleep disturbances (difficulty falling or staying asleep, or restless, unsatisfactory sleep) as symptoms of anxiety (6, 7).

In the late 20th century, there was an increase in armed conflicts, wars, and insurgencies that had a direct impact on civilian populations. During this period, acts of violence and attacks against civilians became common occurrences in global conflicts, resulting in perilous conditions, insecurity, and distress among affected populations. More than a billion young people worldwide reside in countries where armed conflicts, wars, or terrorism are prevalent (8). It is well recognized that the prevalence and severity of mental illnesses in people are significantly impacted by war. In particular, anxiety, depression, and PTSD are the most prevalent mental health problems reported among populations affected by war (9). Anxiety is one of the most prevalent mental health problems for children and adolescents (10), and living in war-affected areas makes the problem more prevalent and complex (11, 12).

Since June 2021, the Tigray People’s Liberation Front (TPLF) forces in Ethiopia have been engaged in an open assault against the Federal Republic Government of Ethiopia in the Amhara and Afar regions. As a consequence, the local population has been subjected to severe economic and social hardships. The invading forces specifically targeted various individuals, including rural farmers, urban residents, teachers, and medical professionals who were not politically involved. Among those affected, children and adolescents face the greatest risk of suffering the detrimental effects of these hostilities, as they must navigate the challenges of normal developmental growth while being exposed to an environment of insecurity and violence, which heightens their vulnerability and susceptibility (13). In addition, the conflict has resulted in 1,035 unwanted pregnancies, multiple cases of rape, and various forms of physical and sexual abuse of women (14). Changes in social dynamics, unemployment, intergroup aggression, poverty, and unstable living conditions are all results of armed conflicts. Therefore, there is a clear relationship between the post-war period and a lower quality of life, which leads to a range of mental health problems, including anxiety (15).

The prevalence of anxiety among adolescents varies widely across different regions, with reported rates ranging from 23.7% to 94.9% (16–19). In 2021, a meta-analysis found a pooled prevalence of 21% (20), while another meta-analysis during the COVID-19 pandemic revealed a pooled prevalence of 26% (21). Additionally, among child and adolescent refugees and asylum seekers, a meta-analysis showed a prevalence of 15.7% (22). Specific studies conducted in various countries and contexts found the following prevalence rates: 18.4% in the USA (23), 34% in Central Europe during the Russian-Ukrainian war in 2022 (24), 37.3% among adolescents in Southwest China following a severe earthquake (25), 37.4% in China during the COVID-19 pandemic (26), 67.1% and 59.1% among students in Malaysia (27, 28), 22.2% and 37% among adolescents in Palestine (29, 30), 24.4% among students in India (31), and 66.2% and 54% in Saudi Arabia (32, 33). Additionally, prevalence rates were reported as 14.9% and 64% among students in Kuwait and Beirut (34, 35), 40.6% and 53.2% among students in Iraq and Pakistan (36, 37), and 41.2% among high school students in Jordan (38). Further studies in Egypt and Ethiopia reported prevalence rates of 21% and 66.7%, respectively, among students (39, 40).

Different variables can affect the prevalence of anxiety among adolescents, as studies have reported. Studies have consistently shown that being of female sex is associated with a higher risk of experiencing anxiety compared to the male sex. While the exact reasons for this gender difference are not fully understood, it is suggested that a combination of biological, hormonal, and sociocultural factors may contribute to this increased vulnerability. Biological factors such as hormonal fluctuations during the menstrual cycle and pregnancy, as well as societal expectations and gender roles, can influence the prevalence of anxiety in women (29, 33, 41, 42). Depression is widely recognized as a significant risk factor for anxiety. The relationship between depression and anxiety is highly intertwined, with individuals often experiencing both conditions simultaneously. Depression involves persistent feelings of sadness, hopelessness, and a loss of interest in activities, while anxiety is characterized by excessive worry, fear, and a sense of restlessness. The coexistence of depression and anxiety can exacerbate the severity of both conditions and create a more challenging clinical picture (41). Having a family history of mental illness has consistently been identified as a risk factor for developing anxiety. Genetic predisposition plays a role in this association, as certain inherited traits or vulnerabilities can increase the likelihood of developing anxiety disorders. Additionally, shared environmental factors within families, such as parenting styles or exposure to stressful events, can contribute to the transmission of anxiety-related behaviors and coping mechanisms from one generation to the next (43–48). Exposure to war situations, such as witnessing the murder of family or friends is another risk factor for developing anxiety, as studies have revealed. The profound trauma and psychological impact of such experiences can lead to the development of anxiety disorders. Exposure to violence, loss, and the constant threat to personal safety can significantly affect the mental well-being of an individual, contributing to the manifestation of anxiety (41, 49, 50). Poor social support has been established as a significant risk factor for anxiety. Social support refers to the availability of emotional, instrumental, and informational assistance from others, including family, friends, and community networks. When individuals lack strong support systems, nurturing relationships, or a sense of belonging, they experience an increased risk of developing anxiety. Social support plays a crucial role in buffering the negative effects of stress and providing a sense of security (48, 51). Individuals who have been subjected to torture or physical abuse are at an elevated risk of developing anxiety. The experience of severe trauma and the resulting psychological wounds can significantly impact mental health, leading to the development of anxiety disorders. Survivors of torture or abuse often require specialized support to address the associated anxiety (52). A history of substance use, such as smoking cigarettes, is linked to an increased likelihood of experiencing anxiety. Substance use can be both a contributing factor and a consequence of anxiety disorders. Some individuals may turn to substances as a form of self-medication to alleviate anxiety temporarily, but in the long run, substance use can exacerbate anxiety and lead to the development of anxiety disorders (53). A high level of perceived stress has been identified as a significant risk factor for anxiety. Perceived stress refers to subjective appraisal of the stressfulness of life events and circumstances of an individual. When individuals consistently perceive their environment as overwhelming and experience a sense of being unable to cope effectively, they are more likely to develop anxiety. Prolonged exposure to chronic stressors can dysregulate the body’s stress response systems, leading to the manifestation of anxiety (54). Studies have shown that older students are more susceptible to experiencing anxiety compared to younger individuals. The transition to adulthood, such as entering higher education or facing new responsibilities in professional or personal domains, can contribute to heightened anxiety levels. The increased pressure to meet academic or career expectations, coupled with the challenges of managing multiple roles and responsibilities, can contribute to anxiety among older students (54). Having a chronic medical illness is also a risk factor, which increases the risk of developing anxiety. Chronic illnesses, such as diabetes, cardiovascular disease, or autoimmune disorders, can have a significant impact on an individual’s physical and psychological well-being. The uncertainty, limitations, and emotional burden associated with managing a chronic condition can contribute to heightened anxiety levels. The presence of anxiety can further complicate the management and prognosis of the underlying medical condition (43).

Adolescent anxiety, if left untreated, can have numerous negative consequences, such as lower academic performance, negative relationships with friends and family, substance abuse, suicidal and homicidal thoughts, and difficulties in finding employment (55, 56). Additionally, untreated anxiety during adolescence increases the likelihood of developing psychiatric problems in adulthood, such as anxiety disorders, major depression, suicide attempts, and psychiatric hospitalizations (57). Anxiety disorders are associated with significant disability and a reduced quality of life due to their high prevalence, chronic nature, and tendency to co-occur with other mental health disorders (58). Moreover, anxiety disorders impose a substantial economic burden, with an estimated cost of €74.4 billion in 2010 across 30 European Union countries (59).

Despite Ethiopia’s recent experience of war and trauma, particularly during the conflict between the Government of Ethiopia and the Tigray People’s Liberation Front (TPLF), there is limited knowledge about the impact of such events on anxiety among high school students in Ethiopia. Therefore, this study aimed to provide baseline data and contribute to the planning, prevention, and treatment of anxiety in adolescents. The research questions addressed in this study are the following: 1. What is the prevalence of anxiety among high school adolescents who have experienced war? 2. Which factors influence the magnitude of anxiety among high school adolescents who have experienced war?

## Materials and methods

### Study area, period, and populations

A school-based cross-sectional study design was conducted from May 23 to June 08 2022, among four secondary and preparatory schools in Woldia town. The schools are Woldia Secondary and Preparatory School, Genetie Secondary and Preparatory School, Mesenado Secondary and Preparatory School, and Millennium Secondary and Preparatory School. Woldia town is located 521 km from Addis Ababa, the Ethiopian capital city, in the Amhara National Regional State of North Wollo zone. There are 46,139 residents in Woldia town; of these, 23,000 are men and 23,139 are women. Approximately 80.49% of the entire population are Ethiopian orthodox Christians followers, while 18.46% are Muslim religion followers. The study included all high school students who attended their class during data collection time and lived in the study area for a minimum of 1 year. Students who were unable to communicate because of acute sickness during the time of data collection and students who lived in the study area for less than 1 year were both excluded from the study.

### Sample size calculations for the study

The sample size of this study was calculated by assuming a single population proportion formula. We calculate sample size using the proportion (P) from the previous study with a proportion of 48.9% (60) with a 95% confidence interval (CI), a margin of error of 4%, and a 10% non-response rate.


$$\begin{array}{c} \text{N}=\frac{{\left(\text{Z}\alpha /2\right)}^{2}\text{p}(\text{p}-1)}{{\text{d}}^{2}}=\frac{{\left(1.96\right)}^{2}.0.489.0.511}{0.04}=599.7+59.9\\ =659.6=600 \end{array}$$

where n = required sample size, p = prevalence, Zα/2 = 1.96 with 95% CI, and d = margin of error = 0.04. The prevalence of anxiety that was taken from a previous study was 48.9% (60). Therefore, the required sample size for this study is 660. Prior to data collection, students were stratified by grade level (grades 9, 10, 11, and 12) considering each grade level as a stratum. The total number of students from the four schools was 5,100, as data obtained from zonal education bureau indicated. Among these, grade 9 students were 1,606, grade 10 were 1,230, grade 11 were 1,179, and grade 12 were 1,085. Then, we made a proportional allocation for each stratum (grade levels), and we drew 208 students from grade 9, 159 students from grade 10, 153 students from grade 11, and 140 students from grade 12. Then we used the computer-generated lottery method by taking the identification number of the student to select study units from each stratum. Finally, the selected students were taken to a hall, and then the questionnaires were administered after orientation.

The questionnaires in this study include both dependent and independent variables. Our dependent variable was anxiety, which was assessed using the nine-item GAD-7 tool. The independent variables include socio-demographic characteristics of study participants, such as age, sex, and grade level, which were collected using structured socio-demographic questions. Clinical factors such as family history of mental illness, having depression, history of chronic medical illness, and trauma-related factors were assessed using a structured yes/no question, and substance-related factors, including khat, tobacco, and alcohol, were assessed using an alcohol, smoking, and substance involvement screening test (ASSIST) tool (61, 62). Psychosocial factors, including social support level, were assessed using the OSLO 3-item social support scale with scores between 3 and 8 classified as poor social support level, a score between 9 and 11 as medium social support level, and a score between 12 and 14 as strong social support level. The perceived stress (PSS) scale, which has 10 items with each item having 0–4 scores and total scores ranging from 0–40, was used to quantify the level of perceived stress. Respondents with scores between 0 and 13 were considered to have low-level perceived stress, those between 14 and 26 with moderate-level perceived stress, and those between 27 and 40 with high-level perceived stress (63).

The data quality of this study was controlled by the appropriate translation of the questionnaire into the local Amharic language. In addition, the data collectors and supervisors received training before the actual data collection period, and each completed questionnaire is checked. The necessary feedback is also offered to the data collectors each morning. Furthermore, the questionnaire was pretested one week before the actual data collection time for 5% (n=33) of students at Meket Secondary and Preparatory School, which is the main study area. The dependent variable assessment tool GAD-7 had a Cronbach alpha of 0.78. Based on the feedback obtained from the pretest, an appropriate modification was made to the questionnaire. The collected data were coded, edited, entered, and checked into the computer using EPI data version 4.6.02 and imported to STATA version 14.0 to generate descriptive statistics like means, standard deviation, frequency, and percentages. To determine an association between dependent and independent variables, adjusted odds ratios were used using logistic regression, and the significance level was determined using a confidence interval of 95%. Bivariable and multivariable logistic regression analyses were used to identify the independent predictors of anxiety. Each independent variable was separately entered into the bivariable analysis. Then, variables with a p-value<0.2 in bivariable analysis were entered into multivariable analysis. Then, variables that showed a statistically significant association with a p-value<0.05 on logistic regression were considered to be the predictors of anxiety.

### Ethical considerations

The ethical clearance for this study was obtained from the institutional review board (IRB) of the University of Gondar. Information about the study was explained to each study participant in the information sheet. Written informed consent from the study participants who were >18 years old and assent for those students<18 years old from their parents, caregivers, or guardians was obtained. The participants were given the right to refuse or discontinue participation at any time, and the chance to ask anything about the study was given. The privacy and confidentiality of study participants’ information were kept anonymously at every stage of data collection by excluding any personal identifiers in the questionnaire. Students were not forced to participate or receive any monetary incentive, and it was solely voluntary-based.

## Results

### Sociodemographic characteristics of the study participants

Data were obtained from 624 high school students with a response rate of 96%. The mean age of the participants was 17.89 ± 1.66. The majority of the study participants (42.5%) were older than 18 years. More than half (53.4%) of the participants were men, and the rest were women. 64.7% of students were from rural areas, and the rest were from urban areas, as shown in **Table 1**.

**Table 1:** Sociodemographic characteristics of the study participants.

Variables	Categories	Frequency	Percent
Age	< 18	246	39.4
	=18	113	18.1
	> 18	265	42.5
Sex	Male	333	53.4
	Female	291	46.6
Residency	Rural	404	64.7
	Urban	220	35.3
Grade level	9	204	32.7
	10	176	28.2
	11	124	19.9
	12	120	19.2
Semester average score in percentage	<70%	355	56.9
	70%–84.5%	224	35.9
	≥ 85%	45	7.2

### Clinical characteristics of the study respondents

**Figure 1:**
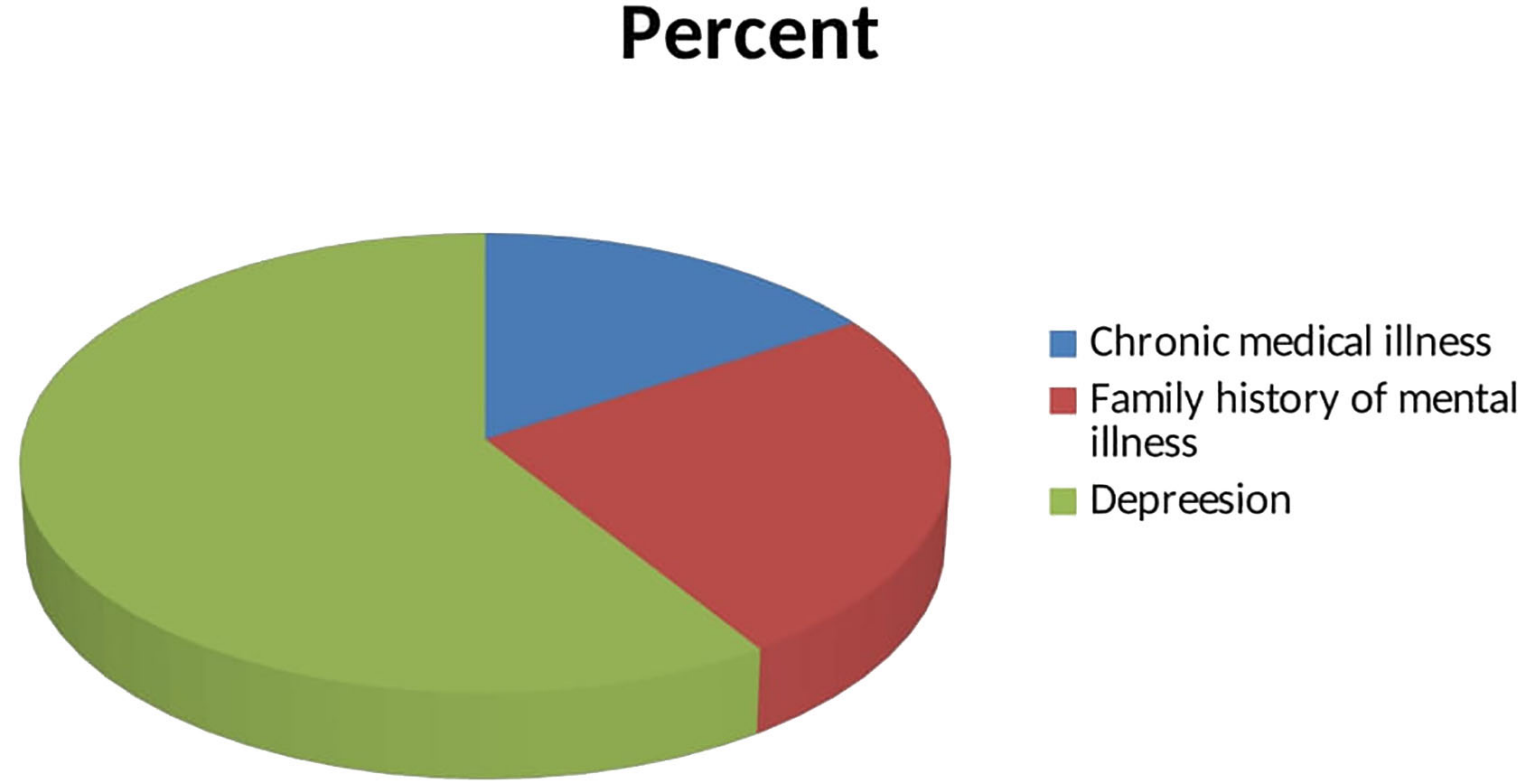
Clinical characteristics of study participants.

Out of the total participants, 11% had a history of chronic medical illness, 17.3% had a family history of mental illness, and 40.9% had depression, as seen in **Figure 1**.

### Substance-use characteristics of the study participants

**Figure 2:**
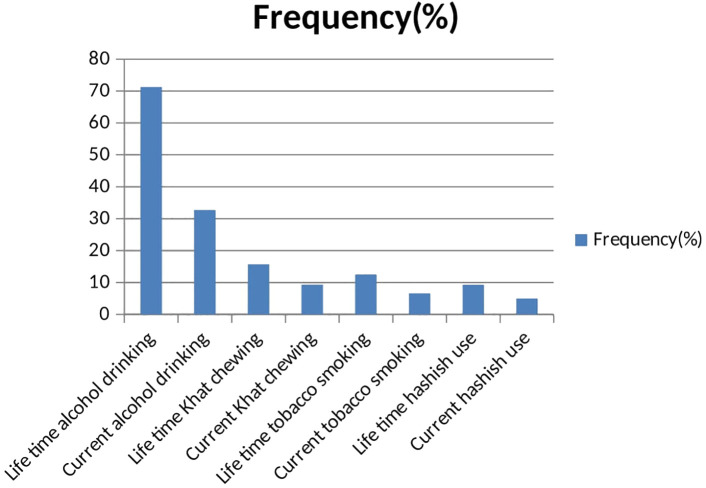
Substance related characteristics of study participants.

Regarding substance use of the participants, 71.3% were drinking alcohol at least once in their lives, whereas students who used khat or cigarettes at least once in their lives were 15.7% and 12.5%, respectively; and 32.7% of them drank alcohol in the last 3 months, as seen in **Figure 2**.

### Trauma type and psychosocial-related characteristics of the study participants

**Figure 3:**
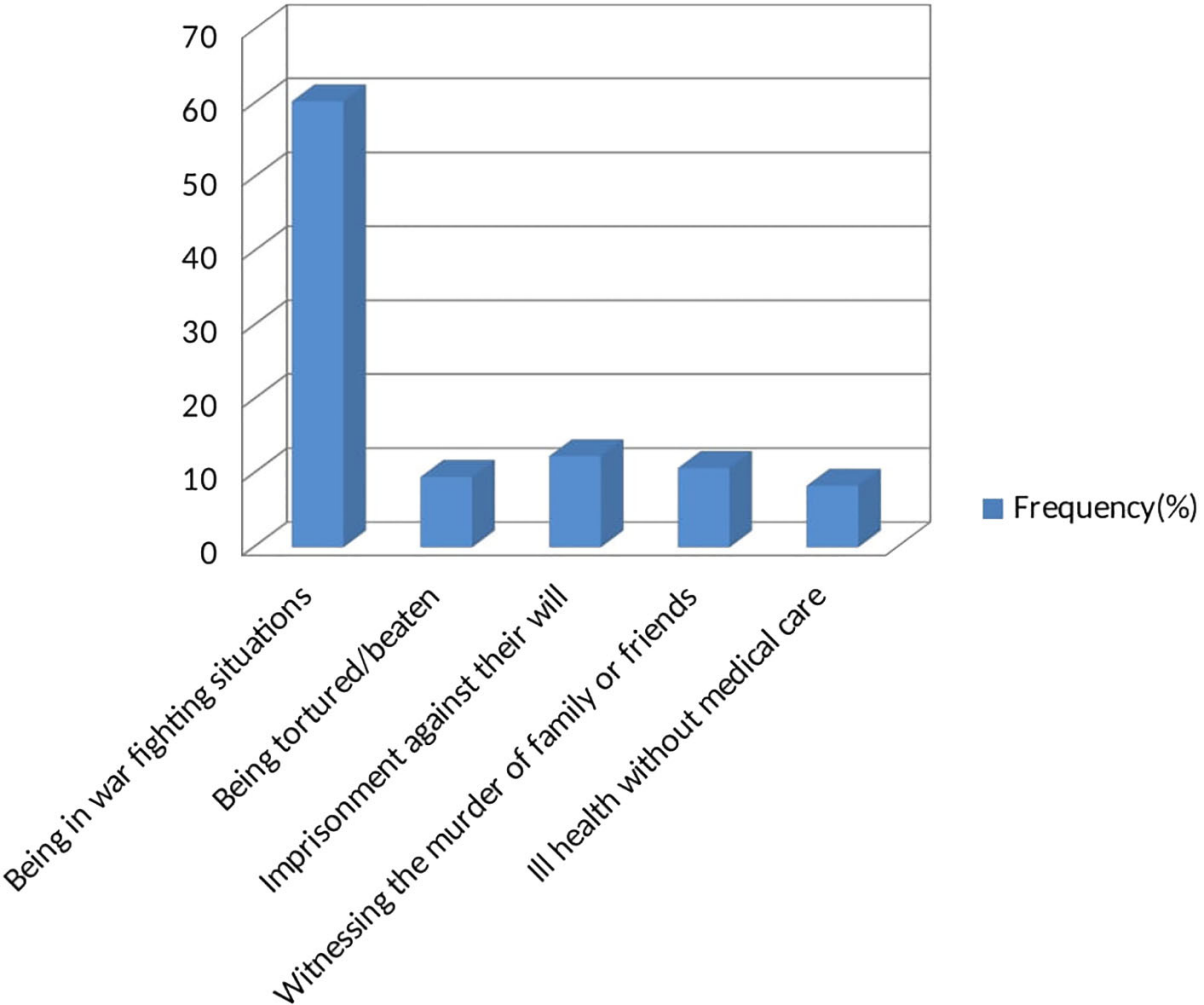
Individual trauma types that the study participants faced.

Among the study participants, 60.3% of them were in a warfighting situation, 9.5% were tortured/beaten, 12.3% had been imprisoned against their will, 10.7% had exposure to traumatic events through witnessing the murder of family or friends, 8.3% had ill health without medical care as seen in **Figure 3**. A total of 30.3% of the students had strong social support, 42.2% had moderate social support, and 27.6% had poor social support. Lastly, 38.8%, 32.5%, and 28.7% of the students had high, moderate, and low levels of perceived stress, respectively.

### The prevalence and associated risk factors of anxiety in this study

In this study, the overall prevalence of anxiety among high school students was 39.7% (95% CI: 35.9%, 43.6%). Having depression, exposure to traumatic events through witnessing the murder of family or friends, having ill health without medical care, witnessing the murder of a stranger, being of female sex, having a family history of mental illness, having chronic medical illness, being older than 18 years, and having poor social support were factors associated with anxiety at p<0.2 in binary logistic regression. Finally, the analysis of the multivariable logistic regression model revealed that having depression, being of female sex, exposure to traumatic events through witnessing the murder of family members or friends, and having a family history of mental illness were found to be significantly associated with anxiety, with a 95% CI and a p-value<0.05, as seen in **Table 2**. Students who had depression were nine times more likely to have anxiety when compared to students who did not have depression (AOR = 9.24, 95% CI: 6.27, 13.64). Students who were exposed to traumatic events such as witnessing the murder of family members or friends were two times more likely to have anxiety than students who did not witness the murder of family members/friends (AOR = 1.93, 95% CI: 1.05, 3.57). Female students had 1.6 times greater anxiety than male students (AOR=1.59, 95% CI: 1.08, 2.36). The students who had a family history of mental illness were 1.7 times more likely to have anxiety than the students who had no family history of mental illness (AOR=1.69, 95% CI: 1.00, 2.85).

**Table 2:** Bivariable and multivariable analyses of factors associated with anxiety among high school students in Woldia town, 2022 (n = 624).

	Category	Anxiety		COR (and 95% CI)	AOR (and 95% CI)
Variables		Yes	No		
Depression	Yes No	178 70	77 299	9.87 (6.79–14.33)	9.24 (6.27–13.64)***
Social support level	Poor Moderate	84 97	88 166	1.73 (1.13–2.65)	1.34 (0.80–2.23)
Family history of mental illness	Yes No	58 190	52 324	1.90 (1.25–2.88)	1.69 (1.00–2.85)*
Sex	Female Male	143 105	148 228	2.09 (1.51–2.90)	1.59 (1.08–2.36)*
Witnessing murder of family/friends	Yes No	37 211	30 346	2.02 (1.21–3.37)	1.93 (1.05–3.57)*
Age	> 18 18	121 42	144 71	1.59 (1.11–2.27)	1.40 (0.91–2.16)
Ill health without medical care	Yes No	14 234	38 338	0.53 (0.28–1.00)	0.52 (0.25–1.10)
Witnessing murder of stranger	Yes No	40 208	80 296	0.71 (0.46–1.08)	0.89 (0.54–1.48)
Having chronic medical illness	Yes No	33 215	36 340	1.44 (0.87–2.39)	1.33 (0.71–2.49)

*= p-value< 0.05, ** = p-value< 0.01, and *** =p value< 0.001.

## Discussion

### Prevalence of anxiety among the study participants

The findings of this study showed that the prevalence of anxiety among high school students was 39.7% (95% CI: 35.9%, 43.6%), which is consistent with similar studies conducted in China (37.3%) (25), Palestine (37%) (30), Iraq (40.6%) (36), and Jordan (41.2%) (38). However, the prevalence in this study was higher than studies conducted in Uganda, India, and Nepal, which reported prevalence rates of 26.6% (41), 24.4% (31), and 10% (64), respectively. The possible reason for this difference could be the difference in assessment tools these studies used. For example, in a study conducted in Uganda, the MINI International Neuropsychiatric Interview for Children and Adolescents (M.I.N.I.-KID) was used; in India, the depression anxiety stress scale (DASS) was used; in Nepal, the Beck anxiety inventory (BAI) was used; but in this study, the GAD-7 tool was used.

Additionally, the difference in the exposure status to war trauma experiences among the study participants in these studies may have contributed to the difference in the prevalence rates; for example, in studies conducted in Uganda and Nepal, their study participants were non-war experiencing school students, but in this study, the study participants experienced war trauma, and different studies revealed that exposure to war trauma experiences is one of the factors that can increase the risk of having anxiety (65–67). Moreover, differences in sample sizes across these studies may also explain the variation in prevalence rates. On the other hand, the prevalence of anxiety in this study was lower than studies conducted in Ethiopia, Beirut, Malaysia, Saudi Arabia, and Pakistan, which reported prevalence rates of 66.7% (43), 64% (35), 67.1% (27), 66.2% (32), and 53.2% (37), respectively. The reason for this difference could be explained by the difference in the assessment tools these studies used. For example, in Malaysia, a 21-item depression, anxiety, and stress scale tool was used; in Pakistan, the Aga Khan University Anxiety and Depression Scale (AKU-ADS) tool was used; in Beirut, a parent-short validated version of the Screen for Childhood Anxiety Related Disorder (SCARED) tool was used; but in this study, the GAD-7 tool was used. Furthermore, the difference in the proportion of female participants in these studies may have influenced the prevalence rates, as being of female sex has been associated with a higher risk of anxiety (33, 38, 47). For example, in a study done in Pakistan, the majority of study participants were female (72%), whereas the number of female participants in this study was 46.6%. Additionally, sample size differences could also contribute to the variations in prevalence rates.

### Associated risk factors for anxiety among the study participants

The result of this study showed that high school students with depression were nine times more likely to have anxiety compared to those without depression. This finding is supported by previous studies (64, 68, 69), suggesting that the neurological abnormalities associated with depression can also lead to the development of anxiety (70, 71). Another factor could be the added difficulty and increased challenge of managing anxious feelings when experiencing symptoms of depression, such as hopelessness and sadness. This can potentially escalate into full-blown anxiety symptoms over time (72), and treating these depressive symptoms is much more advisable in order to halt the progression of anxiety symptoms. Early evaluation and treatment of pre-existing depression among individuals with anxiety can also lead to better outcomes in anxiety treatment. Furthermore, students who had witnessed the murder of family members or friends were found to be twice as likely to have anxiety compared to those who had not experienced such traumatic events. This finding is consistent with previous studies (65–67, 73–75), indicating that trauma can affect brain regions involved in fear regulation and stress responses, increasing the risk of developing anxiety, and this would be more profound when trauma occurred during child and adolescent periods (76). Another possible reason for this may be the enduring impact of traumatic events, which can continue to affect individuals long after the initial trauma. The development of intense feelings of fear and a sense of powerlessness during traumatic experiences can later manifest as anxiety (77). This highlights the crucial importance of effectively managing trauma-related events, particularly in children and adolescents, to address and mitigate the risk of anxiety. The study also revealed that female students had 1.6 times- higher risk of anxiety compared to male students. This finding is supported by a number of studies (33, 38, 41, 48, 65, 78–81) and could be due to the combination of lower stress resistance and higher household workloads typically borne by women compared to men (82, 83). Additionally, the hormonal changes experienced during puberty, including menstruation, may elevate the likelihood of anxiety in women (82). Moreover, the increased prevalence of domestic violence among women compared to men could contribute to increased susceptibility to anxiety (83). Therefore, it is important to address these factors and reduce the burden of household work for women. Lastly, students with a family history of mental illness were 1.7 times more likely to have anxiety compared to those without a family history. This finding is supported by various studies (43, 78, 84, 85) and can be explained by increased stress and potential abuse within a family affected by mental health problems (86). Genetic predisposition (44) and limited caregiver interaction with others due to stigma and caregiving responsibilities may also contribute to a higher risk of anxiety among individuals with a family history of mental illness (87).Therefore, recognizing these potential hazards and taking proactive measures such as seeking professional assistance, adopting relaxation techniques, and adopting a healthier lifestyle can help reduce the risk of anxiety in these individuals (88).

## Conclusions and recommendations

This study revealed that approximately two in five of the high school students in the sample had anxiety symptoms. This study additionally revealed that anxiety symptoms were higher in students with depression, those who had a family history of mental illness, who had been exposed to traumatic events through witnessing the murder of family members or friends, and among female students. Therefore, we recommend that the Ministry of Education work with the Ministry of Health to extend and implement mental health services in high schools and to promote the wellbeing of students in order to prevent anxiety. In addition, we recommend that future researchers work to deal with these population groups as well as identify cause-and-effect relationships among the study variables.

### Strengths and limitations of this study

This study was conducted with some strengths and limitations. To the best of our knowledge, this study was the first to consider high school students who have a history of experiencing war trauma and deal with its effects on the anxiety symptoms of high school students in the study area. Since it was a cross-sectional study, it could be difficult to declare a cause-and-effect relationship between anxiety symptoms and independent factors such as depression and the dependent variable anxiety symptoms; additionally, recall bias may have occurred during data collection since the study collected retrospective data for 1 year. In addition, locally and culturally developed tools were used to assess anxiety and other independent variables in this study; this may have overestimated or underestimated the prevalence rate (89).

## Data availability statement

The original contributions presented in the study are included in the article/supplementary material. Further inquiries can be directed to the corresponding author.

## Ethics statement

This study was ethically approved by The Institutional Review Board (IRB) of the University of Gondar. This study was conducted in accordance with the local legislation and institutional requirements. Written informed consent for participation in this study was provided by the participants’ legal guardians/next of kin.

## Author contributions

MK: Conceptualization, Data curation, Formal analysis, Writing – original draft, Writing – review & editing. TA: Conceptualization, Data curation, Writing – original draft. GN: Conceptualization, Data curation, Writing – original draft. MM: Conceptualization, Data curation, Writing – review & editing. MA: Data curation, Formal analysis, Writing – review & editing. ES: Conceptualization, Writing – original draft, Writing – review & editing. BA: Conceptualization, Data curation, Writing – original draft, Writing – review & editing.

## References

[1] Kapur S. Adolescence: the stage of transition. Horizons holistic education. (2015) 2:233–50.

[2] Hewlett E Moran V. Making mental health count: the social and economic costs of neglecting mental health care. Paris, France: OECD (2014). doi: 10.1787/2074319x

[3] Ogundele MO. Behavioural and emotional disorders in childhood: A brief overview for paediatricians. World J Clin pediatrics. (2018) 7:9. doi: 10.5409/wjcp.v7.i1.9 PMC5803568 29456928

[4] Trivedi JK Gupta PK. An overview of Indian research in anxiety disorders. Indian J Psychiatry. (2010) 52:S210. doi: 10.4103/0019-5545.69234 21836680 PMC3146193

[5] Muris P Mayer B Freher NK Duncan S van den Hout A. Children’s internal attributions of anxiety-related physical symptoms: Age-related patterns and the role of cognitive development and anxiety sensitivity. Child Psychiatry Hum Dev. (2010) 41:535–48. doi: 10.1007/s10578-010-0186-1 PMC2917553 20440551

[6] American Psychiatric Association D. Diagnostic and statistical manual of mental disorders: DSM-5. Washington, DC: American psychiatric association (2013). doi: 10.1176/appi.books.9780890425596

[7] Deardorff J Hayward C Wilson KA Bryson S Hammer LD Agras S. Puberty and gender interact to predict social anxiety symptoms in early adolescence. J Adolesc Health. (2007) 41:102–4. doi: 10.1016/j.jadohealth.2007.02.013 PMC2710300 17577541

[8] Chrisman AK Dougherty JG. Mass trauma: Disasters, terrorism, and war. Child Adolesc Psychiatr Clinics. (2014) 23:257–79. doi: 10.1016/j.chc.2013.12.004 24656579

[9] Steel Z Chey T Silove D Marnane C Bryant RA Van Ommeren M. Association of torture and other potentially traumatic events with mental health outcomes among populations exposed to mass conflict and displacement: a systematic review and meta-analysis. JAMA. (2009) 302:537–49. doi: 10.1001/jama.2009.1132 19654388

[10] Beesdo K Knappe S Pine DS. Anxiety and anxiety disorders in children and adolescents: developmental issues and implications for DSM-V. Psychiatr Clinics. (2009) 32:483–524. doi: 10.1016/j.psc.2009.06.002 PMC3018839 19716988

[11] Slone M Shoshani A. Children affected by war and armed conflict: Parental protective factors and resistance to mental health symptoms. Front Psychol. (2017) 8:1397. doi: 10.3389/fpsyg.2017.01397 28878705 PMC5572511

[12] Cuesta J Leone M. Humanitarian crises and adolescent well-being: knowledge, gaps, and prospects. J Economic Surveys. (2020) 34:3–34. doi: 10.1111/joes.12339

[13] Frounfelker RL Islam N Falcone J Farrar J Ra C Antonaccio CM. Living through war: Mental health of children and youth in conflict-affected areas. Int Rev Red Cross. (2019) 101:481–506. doi: 10.1017/S181638312000017X

[14] Amhara TFI. Ethiopia: summary killings, rape, and looting by Tigrayan forces in Amhara. (2022). London, UK: Amnesty International.

[15] Kashdan TB Morina N Priebe S. Post-traumatic stress disorder, social anxiety disorder, and depression in survivors of the Kosovo War: Experiential avoidance as a contributor to distress and quality of life. J Anxiety Disord. (2009) 23:185–96. doi: 10.1016/j.janxdis.2008.06.006 PMC2667796 18676121

[16] Kolltveit S Lange-Nielsen II Thabet AAM Dyregrov A Pallesen S Johnsen TB. Risk factors for PTSD, anxiety, and depression among adolescents in Gaza. J traumatic stress. (2012) 25:164–70. doi: 10.1002/jts.21680 22522730

[17] Panter-Brick C Eggerman M Gonzalez V Safdar S. Violence, suffering, and mental health in Afghanistan: a school-based survey. Lancet. (2009) 374:807–16. doi: 10.1016/S0140-6736(09)61080-1 PMC2748901 19699514

[18] Pat-Horenczyk R Peled O Daie A Abramovitz R Brom D Chemtob CM. Adolescent exposure to recurrent terrorism in Israel: Posttraumatic distress and functional impairment. Am J Orthopsychiatry. (2007) 77:76–85. doi: 10.1037/0002-9432.77.1.76 17352588

[19] Elbedour S Onwuegbuzie AJ Ghannam J Whitcome JA Hein FA. Post-traumatic stress disorder, depression, and anxiety among Gaza Strip adolescents in the wake of the second Uprising (Intifada). Child Abuse neglect. (2007) 31:719–29. doi: 10.1016/j.chiabu.2005.09.006 17631959

[20] Kowalchuk A Gonzalez SJ Zoorob RJ. Anxiety disorders in children and adolescents. Am Family physician. (2022) 106:657–64. 36521463

[21] Ma L Mazidi M Li K Li Y Chen S Kirwan R. Prevalence of mental health problems among children and adolescents during the COVID-19 pandemic: A systematic review and meta-analysis. J Affect Disord. (2021) 293:78–89. doi: 10.1016/j.jad.2021.06.021 34174475 PMC9711885

[22] Blackmore R Gray KM Boyle JA Fazel M Ranasinha S Fitzgerald G. Systematic review and meta-analysis: the prevalence of mental illness in child and adolescent refugees and asylum seekers. J Am Acad Child Adolesc Psychiatry. (2020) 59:705–14. doi: 10.1016/j.jaac.2019.11.011 31778780

[23] Wu P Goodwin RD Fuller C Liu X Comer JS Cohen P. The relationship between anxiety disorders and substance use among adolescents in the community: specificity and gender differences. J Youth adolescence. (2010) 39:177–88. doi: 10.1007/s10964-008-9385-5 PMC2809931 20084563

[24] Riad A Drobov A Krobot M Antalová N Alkasaby MA Peřina A. Mental health burden of the Russian–Ukrainian war 2022 (RUW-22): anxiety and depression levels among young adults in central Europe. Int J Environ Res Public Health. (2022) 19:8418. doi: 10.3390/ijerph19148418 35886269 PMC9318466

[25] Tang W Lu Y Xu J. Post-traumatic stress disorder, anxiety and depression symptoms among adolescent earthquake victims: comorbidity and associated sleep-disturbing factors. Soc Psychiatry Psychiatr Epidemiol. (2018) 53:1241–51. doi: 10.1007/s00127-018-1576-0 30109368

[26] Sandal RK Goel NK Sharma MK Bakshi RK Singh N Kumar D. Prevalence of depression, anxiety and stress among school going adolescent in Chandigarh. J Family Med primary Care. (2017) 6:405. doi: 10.4103/2249-4863.219988 29302555 PMC5749094

[27] Wahab S Rahman FNA Wan Hasan WMH Zamani IZ Arbaiei NC Khor SL. Stressors in secondary boarding school students: Association with stress, anxiety and depressive symptoms. Asia-Pacific Psychiatry. (2013) 5:82–9. doi: 10.1111/appy.12067 23857842

[28] Latiff LA Tajik E Ibrahim N Bakar ASA Ali Shirin SSA. Psychosocial problem and its associated factors among adolescents in the secondary schools in Pasir Gudang, Johor. Malaysian J Med Health Sci. (2017) 13:35–45.

[29] Qeshta H Hawajri AM Thabet AM. The relationship between war trauma, PTSD, anxiety and depression among adolescents in the Gaza Strip. Health Sci J. (2019) 13:621. doi: 10.21767/1791-809X.1000621

[30] Wagner G Glick P Khammash U Shaheen M Brown R Goutam P. Exposure to violence and its relationship to mental health among young people in Palestine. Eastern Mediterr Health J. (2020) 26:189–97. doi: 10.26719/2020.26.2.189 32141597

[31] Kumar KS Akoijam BS. Depression, anxiety and stress among higher secondary school students of Imphal, Manipur. Indian J Community Med. (2017) 42:94. doi: 10.4103/ijcm.IJCM_266_15 28553025 PMC5427869

[32] Khalid S AG Hasan S AA Ossama AM. Prevalence of depression, anxiety and stress as measured by the depression, anxiety, and stress scale [DASS-42] among secondary school girls in Abha, Saudi Arabia. (2009) 9:140–47. PMC3074779 21509290

[33] Al-Makinah S Al-Aithan Z Al-Quryan A. Prevalence of anxiety and its effect on academic performance among secondary school students in Al-Ahsa city, eastern Saudi Arabia, 2020: cross-sectional study. Ann Clin Analytical Med. (2023) 10:1–13.

[34] Al-Turkait FA Ohaeri JU. Psychopathological status, behavior problems, and family adjustment of Kuwaiti children whose fathers were involved in the first gulf war. Child Adolesc Psychiatry Ment Health. (2008) 2:1–12. doi: 10.1186/1753-2000-2-12 18510770 PMC2423353

[35] Maalouf FT Haidar R Mansour F Elbejjani M El Khoury J Khoury B. Anxiety, depression and PTSD in children and adolescents following the Beirut port explosion. J Affect Disord. (2022) 302:58–65. doi: 10.1016/j.jad.2022.01.086 35085669

[36] Al-Abbudi S. Prevalence of symptoms of depression, anxiety and stress among secondary school students in Baghdad, Iraq. (2018) 10:66257–62.

[37] Ibbad S Baig LA Ahmer Z Shahid F. Prevalence of anxiety and depression in high school students of Karachi, Pakistan. Pakistan J Med Sci. (2022) 38:916. doi: 10.12669/pjms.38.4.5093 PMC9121923 35634611

[38] Malak MZ Khalifeh AH. Anxiety and depression among school students in Jordan: Prevalence, risk factors, and predictors. Perspect Psychiatr Care. (2018) 54:242–50. doi: 10.1111/ppc.2018.54.issue-2 28617949

[39] Al Bahnasy RA Abdel-Rasoul GM Mohamed OA Mohamed NR Ibrahem RA. Prevalence of depression, anxiety, and obsessive–compulsive disorders among secondary school students in Menoufia Governorate, Egypt. Menoufia Med J. (2013) 26:44. doi: 10.7123/01.MMJ.0000429483.48445.DA

[40] Nakie G Segon T Melkam M Desalegn GT Zeleke TA. Prevalence and associated factors of depression, anxiety, and stress among high school students in, Northwest Ethiopia, 2021. BMC Psychiatry. (2022) 22:739. doi: 10.1186/s12888-022-04393-1 36443717 PMC9707065

[41] Abbo C Kinyanda E Kizza RB Levin J Ndyanabangi S Stein DJ. Prevalence, comorbidity and predictors of anxiety disorders in children and adolescents in rural north-eastern Uganda. Child Adolesc Psychiatry Ment Health. (2013) 7:1–11. doi: 10.1186/1753-2000-7-21 23841918 PMC3710504

[42] AlAzzam M Abuhammad S Abdalrahim A Hamdan-Mansour AM. Predictors of depression and anxiety among senior high school students during COVID-19 pandemic: The context of home quarantine and online education. J School Nursing. (2021) 37:241–8. doi: 10.1177/1059840520988548 33563066

[43] Nakie G Segon T Melkam M Desalegn GT Zeleke TA. Prevalence and associated factors of depression, anxiety, and stress among high school students in, Northwest Ethiopia, 2021. BMC Psychiatry. (2022) 22:1–12. doi: 10.1186/s12888-022-04393-1 36443717 PMC9707065

[44] Dachew BA Azale Bisetegn T Berhe Gebremariam R. Prevalence of mental distress and associated factors among undergraduate students of University of Gondar, Northwest Ethiopia: a cross-sectional institutional based study. PLoS One. (2015) 10:e0119464. doi: 10.1371/journal.pone.0119464 25794278 PMC4368633

[45] Dessie Y Ebrahim J Awoke T. Mental distress among university students in Ethiopia: a cross sectional survey. Pan Afr Med J. (2013) 15:1–8. doi: 10.11604/pamj.2013.15.95.2173 24198889 PMC3810159

[46] Cerdá M Sagdeo A Johnson J Galea S. Genetic and environmental influences on psychiatric comorbidity: a systematic review. J Affect Disord. (2010) 126:14–38. doi: 10.1016/j.jad.2009.11.006 20004978 PMC2888715

[47] Mboya IB John B Kibopile ES Mhando L George J Ngocho JS. Factors associated with mental distress among undergraduate students in northern Tanzania. BMC Psychiatry. (2020) 20:1–7. doi: 10.1186/s12888-020-2448-1 31996200 PMC6988278

[48] Serrano IMA Cuyugan AMN Cruz K Mahusay JMA Alibudbud R. Sociodemographic characteristics, social support, and family history as factors of depression, anxiety, and stress among young adult senior high school students in metro Manila, Philippines, during the COVID-19 pandemic. Front Psychiatry. (2023) 14:1225035. doi: 10.3389/fpsyt.2023.1225035 37772068 PMC10525313

[49] Osokina O Silwal S Bohdanova T Hodes M Sourander A Skokauskas N. Impact of the Russian invasion on mental health of adolescents in Ukraine. J Am Acad Child Adolesc Psychiatry. (2023) 62:335–43. doi: 10.1016/j.jaac.2022.07.845 36441074

[50] Kurapov A Danyliuk I Loboda A Kalaitzaki A Kowatsch T Klimash T. Six months into the war: a first-wave study of stress, anxiety, and depression among in Ukraine. Front Psychiatry. (2023) 14:1190465. doi: 10.3389/fpsyt.2023.1190465 37234208 PMC10206008

[51] Mariani R Renzi A Di Trani M Trabucchi G Danskin K Tambelli R. The impact of coping strategies and perceived family support on depressive and anxious symptomatology during the coronavirus pandemic (COVID-19) lockdown. Front Psychiatry. (2020) 11:587724. doi: 10.3389/fpsyt.2020.587724 33281647 PMC7691226

[52] Gormez V Kılıç HN Orengul AC Demir MN Demirlikan Ş Demirbaş S. Psychopathology and associated risk factors among forcibly displaced Syrian children and adolescents. J immigrant minority Health. (2018) 20:529–35. doi: 10.1007/s10903-017-0680-7 29204726

[53] Alenazi SF. Prevalence of depression, anxiety and stress among male secondary school students in Arar city, Saudi Arabia, during the school year 2018: Array. Electronic physician. (2019) 11:7522–8. doi: 10.19082/7522

[54] Kim M-L Shin K. Exploring the major factors affecting generalized anxiety disorder in Korean adolescents: based on the 2021 Korea youth health behavior survey. Int J Environ Res Public Health. (2022) 19:9384. doi: 10.3390/ijerph19159384 35954740 PMC9368270

[55] Hill C Waite P Creswell C. Anxiety disorders in children and adolescents. Paediatrics Child Health. (2016) 26:548–53. doi: 10.1016/j.paed.2016.08.007

[56] Gregory AM Caspi A Moffitt TE Koenen K Eley TC Poulton R. Juvenile mental health histories of adults with anxiety disorders. Am J Psychiatry. (2007) 164:301–8. doi: 10.1176/ajp.2007.164.2.301 17267794

[57] . doi: 10.1111/j.1469-7610.2008.02061.x

[58] Saha S Stedman TJ Scott JG McGrath JJ. The co-occurrence of common mental and physical disorders within Australian families: A national population-based study. Aust New Z J Psychiatry. (2013) 47:754–61. doi: 10.1177/0004867413486841 23630393

[59] Gustavsson A Svensson M Jacobi F Allgulander C Alonso J Beghi E. Cost of disorders of the brain in Europe 2010. Eur Neuropsychopharmacol. (2011) 21:718–79. doi: 10.1016/j.euroneuro.2011.08.008 21924589

[60] Al-Gelban KS. Depression, anxiety and stress among Saudi adolescent school boys. J R Soc Promotion Health. (2007) 127:33–7. doi: 10.1177/1466424007070492 17319315

[61] Gryczynski J Kelly SM Mitchell SG Kirk A O'Grady KE Schwartz RP. Validation and performance of the A lcohol, S moking and S ubstance I nvolvement S creening T est (ASSIST) among adolescent primary care patients. Addiction. (2015) 110:240–7. doi: 10.1111/add.12767 PMC4301997 25311148

[62] Newcombe D Tanielu-Stowers H McDermott R Stephen J Nosa V. The validation of the alcohol, smoking and substance involvement screening test (ASSIST) amongst Pacific people in New Zealand. NZJ Psychol. (2016) 45:31–40.

[63] Sharma R Bansal P Chhabra M Bansal C Arora M. Severe acute respiratory syndrome coronavirus-2-associated perceived stress and anxiety among Indian medical students: A cross-sectional study. Asian J Soc Health Behavior. (2021) 4:98. doi: 10.4103/shb.shb_9_21

[64] Bhandari M. Anxiety and depression among adolescent students at higher secondary school. Bibechana. (2017) 14:103–9. doi: 10.3126/bibechana.v14i0.16019

[65] Acarturk C McGrath M Roberts B Ilkkursun Z Cuijpers P Sijbrandij M. Prevalence and predictors of common mental disorders among Syrian refugees in Istanbul, Turkey: a cross-sectional study. Soc Psychiatry Psychiatr Epidemiol. (2021) 56:475–84. doi: 10.1007/s00127-020-01941-6 32789561

[66] Husain F Anderson M Cardozo BL Becknell K Blanton C Araki D. Prevalence of war-related mental health conditions and association with displacement status in postwar Jaffna District, Sri Lanka. JAMA. (2011) 306:522–31. doi: 10.1001/jama.2011.1052 21813430

[67] Tinghög P. Mental ill-health, trauma and adverse post-migratory experiences among refugees from Syria in Sweden: Petter Tinghög. Eur J Public Health. (2017) 27:ckx187–26. doi: 10.1093/eurpub/ckx187.126

[68] Islam MS Rahman ME Moonajilin MS van Os J. Prevalence of depression, anxiety and associated factors among school going adolescents in Bangladesh: Findings from a cross-sectional study. PloS One. (2021) 16:e0247898. doi: 10.1371/journal.pone.0247898 33793610 PMC8016317

[69] Yu Y Liu J Skokauskas N Liu F Zhang L Teng T. Prevalence of depression and anxiety, and associated factors, among Chinese primary and high school students: A cross-sectional, epidemiological study. Asia-Pacific Psychiatry. (2023) 15:e12523. doi: 10.1111/appy.12523 36596718

[70] Kovner R Oler JA Kalin NH. Cortico-limbic interactions mediate adaptive and maladaptive responses relevant to psychopathology. Am J Psychiatry. (2019) 176:987–99. doi: 10.1176/appi.ajp.2019.19101064 PMC7014786 31787014

[71] Etkin A Schatzberg AF. Common abnormalities and disorder-specific compensation during implicit regulation of emotional processing in generalized anxiety and major depressive disorders. Am J Psychiatry. (2011) 168:968–78. doi: 10.1176/appi.ajp.2011.10091290 21632648

[72] Available online at: https://www.verywellhealth.com/depression-and-anxiety-signs-symptoms-and-treatment-5191284.

[73] De Jong JTVM Komproe IH Van Ommeren M. Common mental disorders in postconflict settings. Lancet. (2003) 361:2128–30. doi: 10.1016/S0140-6736(03)13692-6 12826440

[74] Priebe S Bogic M Ajdukovic D Franciskovic T Galeazzi GM Kucukalic A. Mental disorders following war in the Balkans: a study in 5 countries. Arch Gen Psychiatry. (2010) 67:518–28. doi: 10.1001/archgenpsychiatry.2010.37 20439833

[75] Cardozo BL Bilukha OO Crawford CAG Shaikh I Wolfe MI Gerber ML. Mental health, social functioning, and disability in postwar Afghanistan. JAMA. (2004) 292:575–84. doi: 10.1001/jama.292.5.575 15292083

[76] Available online at: https://www.urmc.rochester.edu/news/publications/neuroscience/researchers-reveal-how-trauma-changes-the-brain.

[77] Available online at: https://www.steadyhealth.com/articles/trauma-and-anxiety-whats-the-connection.

[78] Melkam M Nenko G Demilew D. Common mental disorders and associated factors among high school students in Debre Markos Town, Northwest Ethiopia: an institutional-based cross-sectional study. BMJ Open. (2022) 12:1–9. doi: 10.1136/bmjopen-2021-059894 PMC9639070 36332965

[79] Ramón-Arbués E Gea-Caballero V Granada-López JM Juárez-Vela R Pellicer-García B Antón-Solanas I. The prevalence of depression, anxiety and stress and their associated factors in college students. Int J Environ Res Public Health. (2020) 17:7001. doi: 10.3390/ijerph17197001 32987932 PMC7579351

[80] Crocetti E Hale WW Dimitrova R Abubakar A Gao C-H Pesigan IJA eds. Generalized anxiety symptoms and identity processes in cross-cultural samples of adolescents from the general population. London, UK: Springer (2015). doi: 10.1007/s10566-014-9275-9

[81] Pieh C Budimir S Probst T. The effect of age, gender, income, work, and physical activity on mental health during coronavirus disease (COVID-19) lockdown in Austria. J psychosomatic Res. (2020) 136:110186. doi: 10.1016/j.jpsychores.2020.110186 PMC7832650 32682159

[82] Gebremedhin HT Bifftu BB Lebessa MT Weldeyes AZ Gebru TT Petrucka P. Prevalence and associated factors of psychological distress among secondary school students in Mekelle city, Tigray region, Ethiopia: Cross-sectional study. Psychol Res Behav Manage. (2020) 473–80. doi: 10.2147/PRBM.S252779 PMC7250291 32547269

[83] Pengpid S Peltzer K. Prevalence and associated factors of psychological distress among a national sample of in-school adolescents in Morocco. BMC Psychiatry. (2020) 20:1–11. doi: 10.1186/s12888-020-02888-3 32993597 PMC7526246

[84] Alvi T Assad F Ramzan M Khan FA. Depression, anxiety and their associated factors among medical students. J Coll Physicians Surg Pak. (2010) 20:122–6. 20378041

[85] Nakie G Melkam M Desalegn GT Zeleke TA. Prevalence and associated factors of social phobia among high school adolescents in Northwest Ethiopia, 2021. Front Psychiatry. (2022) 13:949124. doi: 10.3389/fpsyt.2022.949124 36387008 PMC9640733

[86] Available online at: https://www.amenclinics.com/blog/5-ways-your-family-history-affects-your-mental-health/.

[87] Yimam K Kebede Y Azale T. Prevalence of common mental disorders and associated factors among adults in Kombolcha Town, Northeast Ethiopia. J Depression Anxiety. (2014) 1:007. doi: 10.4172/2167-1044

[88] Available online at: https://sickday.com/the-role-of-family-history-in-anxiety/#:~:text=When%20multiple%20members%20of%20a%20family%20have%20asioE.

[89] Veronese G Mahamid F Bdier D Obaid H Cavazzoni F. The development and validation of the Palestinian children's traumatic events checklist in a war-torn environment. BMC Psychiatry. (2024) 24:1–11. doi: 10.1186/s12888-024-05731-1 38570753 PMC10988932

